# Cognitive status correlates of subclinical action tremor in female carriers of FMR1 premutation

**DOI:** 10.3389/fneur.2024.1401286

**Published:** 2024-06-06

**Authors:** Danuta Z. Loesch, Anna Atkinson, Deborah A. Hall, Flora Tassone, Paige Stimpson, Elsdon Storey

**Affiliations:** ^1^School of Psychology and Public Health, La Trobe University, Bundoora, VIC, Australia; ^2^Department of Neurological Sciences, Rush University Medical Centre, Chicago, IL, United States; ^3^Department of Biochemistry and Molecular Medicine, School of Medicine, University of California, Davis, Davis, CA, United States; ^4^MIND Institute, University of California Davis Medical Centre, Davis, CA, United States; ^5^Wellness and Recovery Centre, Monash Medical Centre, Clayton, VIC, Australia; ^6^Department of Medicine (Neuroscience), Alfred Hospital Campus, Monash University, Melbourne, VIC, Australia

**Keywords:** fragile X premutation, tremor, executive functioning, motor scores, FXTAS, correlations

## Abstract

**Background:**

There is evidence for a significant excess of kinetic upper limb tremor in non-FXTAS female FMR1 premutation carriers. The present study explores the possibility that this tremor is associated with various other features reminiscent of those occurring in syndromic FXTAS.

**Sample/methods:**

This study analyzed the data from an Australian cohort of 48 asymptomatic premutation women. We utilized spiral drawings from CRST, representing action tremor; the CRST total tremor; and ICARS- kinetic tremors/cerebellar ataxia scales. Cognitive tests (involving executive functioning) included SDMT, TMT, two subtests of the WAIS-III: MR and Similarities. Spearman Rank correlations assessed the relationships between the above measures, and the Chi-square tested hypothesis about the association between the white matter hyperintensities (*wmhs*) in the splenium of corpus callosum assessed from MR images and spiral drawings scores.

**Results:**

The spiral drawing scores were significantly correlated with all three non-verbal cognitive test scores, and with the CRST scores; the latter correlated with all four cognitive test measures. Similarities (verbal) scores correlated with CRST, ICARS, and with the remaining cognitive scores. Ordered spiral scores’ categories were significantly associated with the degree of splenium involvement.

**Conclusion:**

This study showed that, in non-FXTAS premutation female carriers, sub-symptomatic forms of kinetic tremor were associated with a broader motor, and cognitive (especially executive) dysfunction.

## 1 Introduction

Premutation in the FMR1 X-linked gene is characterized by the presence of an expanded CGG repeat in the non-coding section, ranging from 55 to 200 (1). Since this mutation is unstable across generations if transmitted through the female parent, the children of premutation mothers are at a higher risk of further (>200) CGG expansions, known as full mutations (2). While full mutations were commonly recognized as being the cause of Fragile X syndrome (FXS), a severe neurodevelopmental disorder, the premutations were initially believed to have no effect on the phenotype (3).

The earliest breakthroughs came with the discovery of causal association between premutation and an early menopause (4), and the reports of the occurrence of FXS-like physical feature and mild cognitive impairments in some adult premutation carriers (5). These were followed by a discovery, in 2001, of a severe, late-onset progressive neurodegenerative disorder, characterized by (predominantly intention) tremors, cerebellar gait ataxia, executive dysfunction, and parkinsonism in some case (6). This disorder, named Fragile X-Associated Tremor-Ataxia Syndrome (FXTAS), occurs in nearly half of aging male, but only in approximately 16% of female premutation carriers due to X-inactivation (7, 8). This syndrome is associated with white matter degeneration: in males, it predominantly affects cerebellar peduncles, where MCP sign has been regarded as a major diagnostic radiological feature (9, 10). However, in FXTAS females, MCP sign is rare, since degeneration occurs more commonly in the splenium of corpus callosum, it is recognized, according to the latest criteria of FXTAS, as minor diagnostic feature (10). According to these criteria, definite diagnosis of FXTAS requires notable presence of one clinical and one radiological characteristic (major) features, whereas two clinical major, or one clinical major and one radiological minor are required for ‘probable’ diagnosis. FXTAS may be suspected (‘possible’) if two clinical features (one major and one minor) are present. Consequently, carrier females usually fall into the two latter categories (probable or possible), which makes decision concerning the level and type of neurological involvement particularly difficult.

More recently, attention has been drawn to the existence of low-symptomatic/ mono-symptomatic forms of neural involvement, especially in female premutation carriers (11). In this study the predominant, often isolated finding was kinetic tremor, which generally progressed over the period of approximately 10 years. Other rare publications also reported apparently isolated tremors in the non-FXTAS female carriers (12, 13), or provided a review of the spectrum of various types of tremors among carriers with or without FXTAS (14).

The latest evidence for a significant excess of kinetic upper limb tremor in premutation carriers compared with controls was based on objective data from two independent samples of non-FXTAS adult females, based on A and B spiral drawing scores (15). In the present study we aimed to explore the possibility that the apparently isolated tremor, occurring in a significant proportion of non-FXTAS female carriers, may in fact be associated with some other features reminiscent of impairments typically occurring in a diagnosable FXTAS.

## 2 Materials and methods

### 2.1 Sample description

This study included the data from an Australian cohort of FMR1 premutation carrier women, who had originally been ascertained by cascade testing, either through their children diagnosed with FXS, or more distant relatives diagnosed as FXS or carrying premutation alleles. Except for one (Thai) female, all participants were white Caucasians. The data applied in this study was collected between 2008 and 2010, within the projects supported by research grants from the National Health and Medical Research Council of Australia (NHMRC) and the National Institute of Health, US, to DZL & ES. All the participants provided informed consent according to protocols approved by the La Trobe University and Monash University Human Research Ethics Committees.

Selected data from this cohort have already been included in earlier publications (reviewed in: (11)), and the newly derived scores for drawing spirals have been used in the latest report (Hall submitted). CGG repeat sizing to establish premutation status was conducted at the MIND Institute, University of California Davis Medical Center, Sacramento, CA, USA using Southern blot analysis (16) and PCR amplification of genomic DNA (17).

### 2.2 Data scoring and analysis

In this study, we have utilized several of these test results from 48 participants from this cohort. The data on upper limb tremor, which was represented by the scores for Drawing A + B (spirals) rated 0–4 each, as in the Clinical Rating Scale for Tremor, CRST (18), was correlated with the total CRST, and the total International Cooperative Ataxia Rating Scale, ICARS (19) scores. Cognitive status correlates included The Symbol Digit Modalities Test, SDMT (20), which is a measure of psychomotor processing speed; The Trial Making test, TMT-A and TMT-B, measuring psychomotor speed, visual search, and attention (21); TMT-B also measures other components of executive control, including set-shifting and cognitive flexibility, and TMTB-TMTA scores, to adjust for potential motor impairments bias. We also included the scaled scores of two subsets of the Wechsler Adult Intelligence Scale, WAIS-III, Third Edition (22): Matrix Reasoning, MRsc, providing a measure of non-verbal reasoning, and Similarities, Sim.Sc, assessing logical thinking, verbal concept formation and verbal (abstract) reasoning.

Brain MRI scans available in a subsample of 35 carriers were acquired using a 1.5 Tesla Siemens scanner located at St. Vincent’s and Alfred Hospitals in Melbourne. Scans were captured using turbo spin-echo 2 dimensional (i) proton-density with T2 weighting (TR 3500, TE 13/103) and/or (ii) fluid-attenuated inversion recovery (FLAIR) (TR 9000, TE 90, TI 2500) axial images with a 5 mm slice thickness or 3D FLAIR (TR 5800, TE 315, TI 2200) with 1 mm voxel size. For this particular study the focus was on the *wmhs* in the cerebellum presenting us MCP sign, and in corpus callosum presenting as Splenium sign.

#### 2.2.1 Statistical analysis

Spearman’s Rank correlations were used to assess the relationships between the A + B drawings and the other motor and cognitive scores, with age correction applied wherever appropriate. Chi square Fisher test was applied to assess the relationship between the binomial representation of A + B drawings (0 or > 0 categories) and splenium (present or absent), and direct linear association between ordered categories, using SPSS package Version 29.0.1.0.

## 3 Results

The age of included participants ranged from 26 (one individual) to 77 (Mean = 50.44, SD = 10.82), but sample size may be less for individual pairs of variables in correlation analysis. None of the participants met the diagnostic criteria for either definite or probable FXTAS. Moreover, none reported tremor or any other motor or self-identified cognitive impairments, and all the participants considered themselves to be in good general health. They were examined jointly by two experienced neurologists, and a neuropsychologist, using an array of motor and cognitive tests. CGG repeat number ranged from 56 to 175 (Mean = 84.4; SD = 21.17); Prorated IQ was 105.55 (SD = 12.33). Detailed description of the results of cognitive tests, including the ones used in this study (as listed in the Data section), based on the same sample, was already presented in our earlier publications (23, 24). These results showed that the mean scores for TMT and SDMT were both suggestive of mild impairment, but the difference with normative values did not exceed one standard deviation.

**Figure 1:**
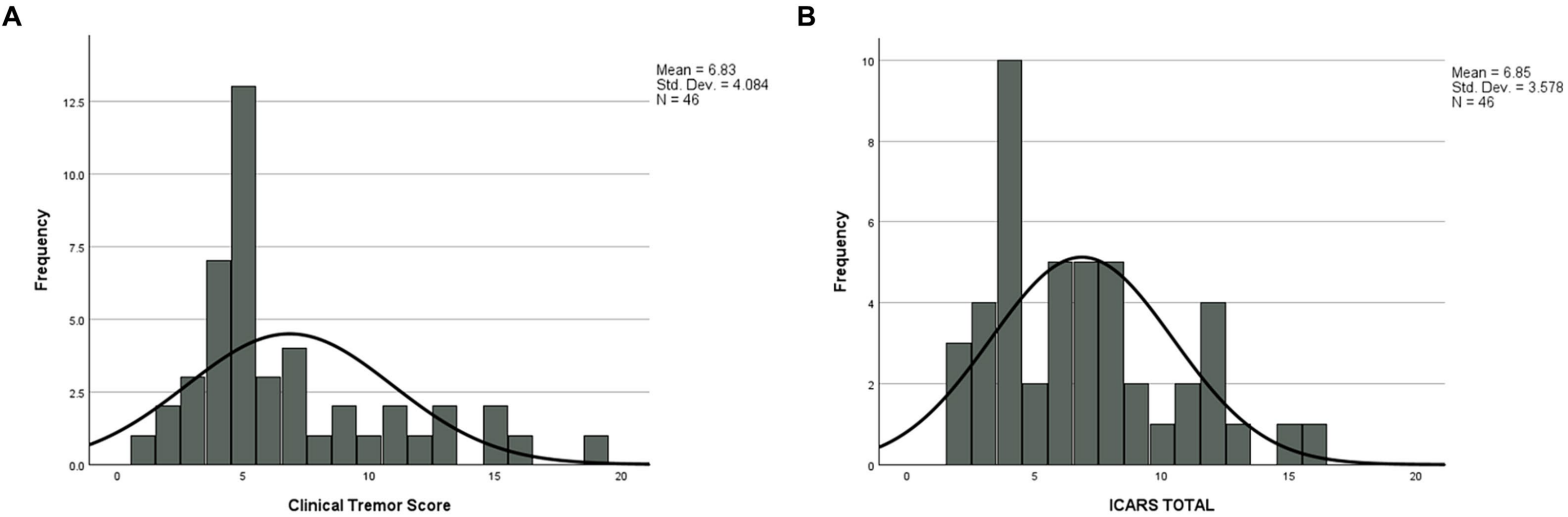
Frequency distributions for **(A)** Clinical tremor and **(B)** ICARS scores.

**Figure 2:**
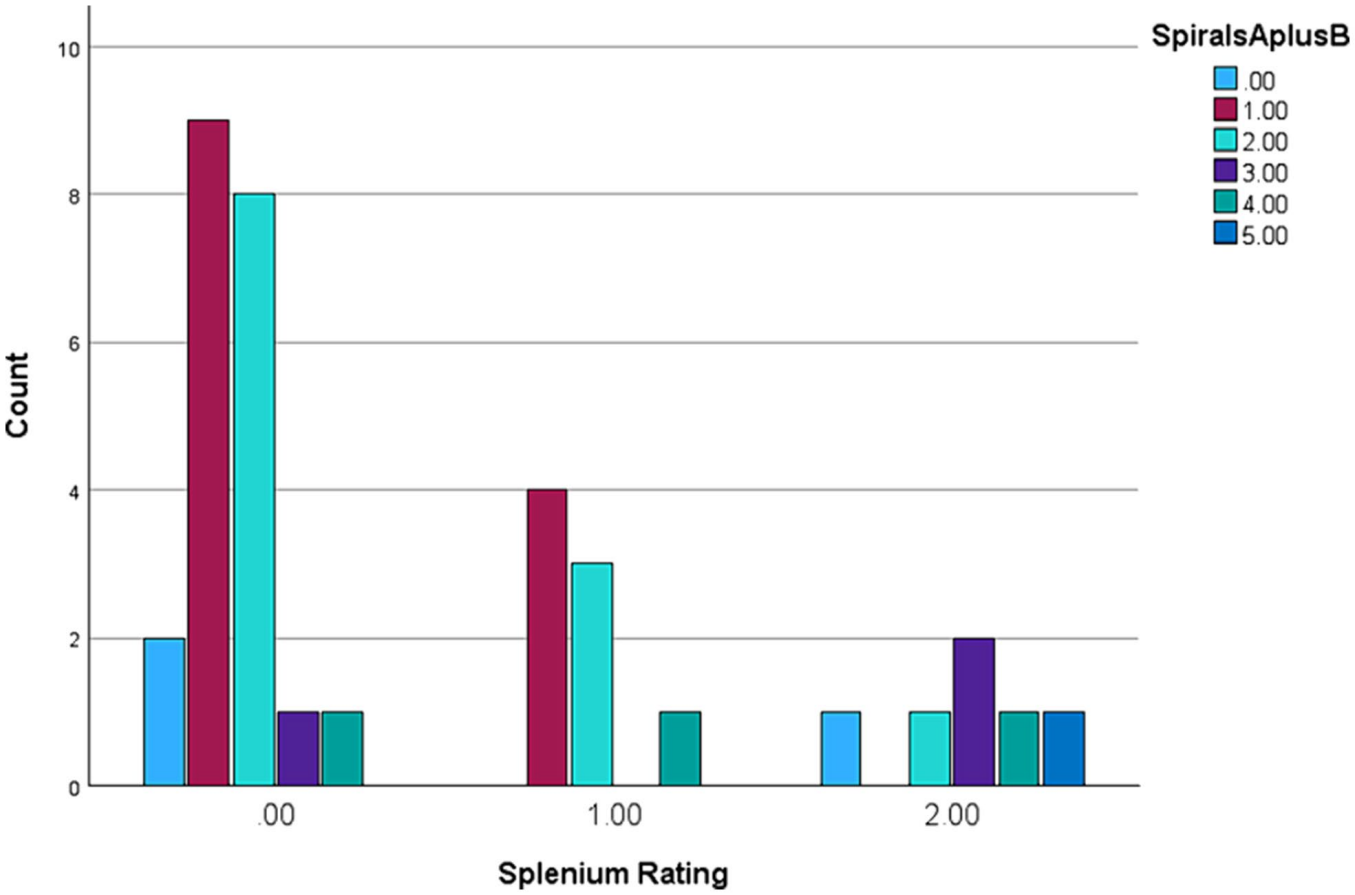
Spiral A + B categories ranged 0–5 against Splenium *wmhs* 0–2 ranking in a subsample of 35 female premutation carriers.

The results in Table 1 show Spearman’s rank correlations between cognitive-executive measures and motor scale scores. The A + B drawing scores are significantly correlated with SDMT, both TMTB and TMTB-A, and MRsc (if one-tailed p is used) - all four tests tapping into higher cognitive (executive) functioning. More predictably, the sum of drawing scores was closely associated with the (total) CRST score, which was also highly correlated with all the cognitive test scores included. Although the correlation between the spiral A + B drawing scores and ICARS has been insignificant, frequency distributions (Figures 1A,B) show that an excess of individual scores above the corresponding means appears greater for the ICARS than for CRST, with the average of 6.85, which exceeds the normal value of 4.07 by more than one SD of 2.19 (25). SIM scale scores did not show significant correlations with the A + B drawing scores, but they were highly correlated with CRST, ICARS, as well as with the other cognitive scores included here There was also a significant relationship between the ordered categories of *wmhs* in the splenium seen on MR images and A + B drawing scores in a small subsample of 35 carriers (two-sided *p* = 0.029 for linear-by-linear association), illustrated in Figure 2. No MCP sign was recorded in this subsample.

**Table 1:** Spearman’s rank correlations between cognitive-executive measures and motor scale scores in female premutation carriers.

Variables			Spirals A + B	TMT B	TMTB - TMTA	ICARS TOTAL	Clinical Tremor	Sim.Sc	MR.Sc	SDMT *Z* score
Spirals A + B	Correlation								
	*p*-value								
	df									TMT B	Correlation	0.334							
	*p*-value	0.031							
	df	40								TMTB - TMTA	Correlation	0.324	0.924						
	*p*-value	0.039	<0.001						
	df	39	41							ICARS TOTAL	Correlation	0.074	0.675	0.579					
	*p*-value	0.628	<0.001	<0.001					
	df	43	40	39						Clinical Tremor	Correlation	0.490	0.512	0.384	0.496				
	*p*-value	<0.001	<0.001	0.013	<0.001				
	df	43	40	39	43					Sim.Sc	Correlation	−0.230	−0.544	−0.525	−0.318	−0.307			
	*p*-value	0.133	<0.001	<0.001	0.035	0.040			
	df	42	42	41	42	45				MR.Sc	Correlation	−0.263	−0.607	−0.609	−0.419	−0.441	0.531		
	*p*-value	0.081	<0.001	<0.001	0.004	0.002	<0.001		
	df	43	42	41	43	46	48			SDMT Z SCORE	Correlation	−0.382	−0.563	−0.475	−0.483	−0.474	0.302	0.422	
	*p*-value	0.013	<0.001	0.001	0.001	0.001	0.039	0.003	-
	df	40	41	40	40	43	45	45	

Age was significantly correlated with Spirals A +B, TMT B, TMTB -TMTA and ICARS Total. Correlations including these variables have been corrected for age. All *p*-values are two-tailed.

## 4 Discussion

The results of our earlier study led to the speculation that kinetic (action) tremor, as assessed using spiral drawings scale, may occur - as an isolated trait - long before development of other FXTAS-like, especially cognitive, changes (26). Here we intended to verify this conjecture by considering the same tremor measures in context with several other motor and cognitive test scores relevant to FXTAS symptomatology, in the Australian cohort (Cohort2 in: 26) of asymptomatic/low symptomatic female carriers. More specifically, we have demonstrated that this apparently isolated feature has been related to the CTRS scale, which assesses several forms of tremors, and to the total ICARS, representing aspects of cerebellar involvements beyond kinetic tremors. Since the average IQ representing general cognition was within the population norm in our sample, we have selected the measures among the already available battery in our cohort, which represent higher cognitive (executive) skills known to be impaired in this category of carriers (27, 28). Significant correlations of all the non-verbal cognitive scale measures with the sum of spiral drawing scores indicate broader neural involvement in apparently asymptomatic carriers. In support of this concept, we also encountered highly significant association of these scores with the total CRST, which assesses the major forms of tremors, in addition to the kinetic one involved in the drawings. Notably, this scale was also significantly correlated with all non-verbal and verbal cognitive scale scores. A broader neural involvement beyond kinetic/action tremor in our sample of premutation carriers is also indicated by frequency distributions of CRST and ICARS scores. They show that an excess of the scores above the population average is notably greater for the latter, even though kinetic tremor contributes only a proportion to the ICARS total, among the other domains of cerebellar dysfunction, such as gait ataxia, and speech and oculomotor changes. A concept of broader neural involvement has been further supported by the finding of linear association between the degree of white matter hyperintensities in the splenium of corpus callosum, and the spiral scores ratings. It is important to note that this splenial change is not only a marker of neurodegeneration, but is also a major diagnostic feature in female FXTAS.

Our earlier results of correlations between some other motor and cognitive test scores, and major psychiatric measures conducted in the same female cohort, showed that more than one category of clinical manifestation reflecting cerebellar changes – motor, higher cognitive, and neuropsychiatric - may be simultaneously affected by premutation carriage across a broad age range in asymptomatic female carriers (23, 24); a similar trend has been reported in an independent sample of carrier males (29). The earlier autopsy results, showing the presence of intranuclear inclusions in both FXTAS and non-FXTAS female carriers provided evidence for an early neurodegenerative process, presumably underpinning the observed relationships between (largely subclinical) changes reminiscent of those that are characteristic of a diagnosable FXTAS (30).

Clearly, systematic follow-up studies of low-symptomatic/ subclinical tremors and associated features in premutation females are required to understand the nature of these changes, the risks of conversion to syndromic forms, and to what extent they reflect an early stage of neurodegeneration. In our earlier study of a subsample of 19 female carriers from the present cohort, who showed the elevation of at least one of the three motor scores (ICARS, CRST, UPDRS), the repeated testing in 13 available females after 10 years showed that half of these females developed a diagnosable FXTAS, while another half showed (usually slow) progression in a single feature, most often evident for tremor. Similar studies, including identification of possible biomarkers of progression, could reveal possible mechanisms determining diverse clinical outcomes.

Incidentally, we note that the failure to recognize symptoms of motor involvement despite evident signs may itself be a feature of impaired higher cognitive functioning. Furthermore, it is worth noting that the scores from all three tests tapping into different subdomains of this functioning are very highly intercorrelated.

This study has some obvious limitations. The relatively small sample size implies low power, thus limiting the number of significant correlations to close relationships. However, the number and consistency of close associations between the spiral scores and the range of cognitive scores have provided sufficient evidence that, in non-FXTAS premutation female carriers, the subclinical, or mild forms of kinetic tremors, are linked with a broader motor, and especially executive functioning, involvements, as well as some FXTAS-like neurodegenerative changes.

Potential bias related to use of retrospective data in our study has been minimized by several factors. The data was collected from a single, and more recently reviewed (11) cohort. Furthermore, their neurological/cognitive status was assessed by the same team consisting of two neurology specialists (ES &DL) with high inter-rated compatibility and a psychologist; we also collected a comprehensive information about their general health status and other risk factors. The raters were blinded for the size of CGG expansion, but not for the genetic status, since the great majority of participants had been recruited through their FXS-affected offspring. However, this could potentially introduce some bias to the assessment in comparative rather than relationship analyses.

## 5 Conclusion

Despite some limitations, our preliminary data obtained from a sample of apparently asymptomatic female premutation carriers has shown that a subclinical kinetic tremor is associated with mild cognitive impairment involving executive functioning, and a marker of neurodegeneration - white matter hyperintensities in the splenium of corpus callosum prevalent in FXTAS females. It appears that there is a potential, in the carriers with broader neural involvement, for diverse clinical outcomes, ranging from asymptomatic to severe, and further studies of common pathological mechanisms underlying this constellation of changes, are recommended.

## Data availability statement

The raw data supporting the conclusions of this article will be made available by the authors, without undue reservation.

## Ethics statement

The studies involving humans were approved by La Trobe University Human Research Ethics Committee and Monash University Human Research Ethics Committee. The studies were conducted in accordance with the local legislation and institutional requirements. The participants provided their written informed consent to participate in this study.

## Author contributions

DL: Conceptualization, Funding acquisition, Investigation, Methodology, Project administration, Resources, Supervision, Validation, Writing – review & editing, Writing – original draft. AA: Data curation, Formal analysis, Project administration, Visualization, Writing – review & editing. DH: Conceptualization, Validation, Writing – review & editing. FT: Data curation, Methodology, Validation, Writing – review & editing. PS: Data curation, Project administration, Writing – review & editing. ES: Conceptualization, Funding acquisition, Investigation, Methodology, Project administration, Resources, Supervision, Validation, Writing – original draft, Writing – review & editing.

## References

[1] Hagerman PJ Hagerman RJ. The fragile-X premutation: a maturing perspective. Am J Hum Genet. (2004) 74:805–16. doi: 10.1086/386296, PMID: 15052536 PMC1181976

[2] Loesch D Hagerman R. Unstable mutations in the FMR1 gene and the phenotypes. Adv Exp Med Biol. (2012) 769:78–114. doi: 10.1007/978-1-4614-5434-2_6, PMID: 23560306 PMC4124039

[3] Pembrey ME Winter RM Davies KE. A premutation that generates a defect at crossing over explains the inheritance of fragile X mental retardation. Am J Med Genet. (1985) 21:709–17. doi: 10.1002/ajmg.1320210413, PMID: 4040705

[4] Loesch DZ Hay DA Mulley J. Transmitting males and carrier females in fragile X–revisited. Am J Med Genet. (1994) 51:392–9. doi: 10.1002/ajmg.1320510418, PMID: 7943005

[5] Sherman SL. Premature ovarian failure in the fragile X syndrome. Am J Med Genet. (2000) 97:189–94. doi: 10.1002/1096-8628(200023)97:3<189::AID-AJMG1036>3.0.CO;2-J 11449487

[6] Hagerman RJ Leehey M Heinrichs W Tassone F Wilson R Hills J. Intention tremor, parkinsonism, and generalized brain atrophy in male carriers of fragile X. Neurology. (2001) 57:127–30. doi: 10.1212/WNL.57.1.127, PMID: 11445641

[7] Hall DA Robertson-Dick EE O'Keefe JA Hadd AG Zhou L Berry-Kravis E. X-inactivation in the clinical phenotype of fragile X premutation carrier sisters. Neurol Genet. (2016) 2:e45. doi: 10.1212/NXG.0000000000000045, PMID: 27066582 PMC4817899

[8] Hagerman RJ Leavitt BR Farzin F Jacquemont S Greco CM Brunberg JA. Fragile-X-associated tremor/ataxia syndrome (FXTAS) in females with the FMR1 premutation. Am J Hum Genet. (2004) 74:1051–6. doi: 10.1086/420700, PMID: 15065016 PMC1181968

[9] Leehey MA Hall DA Liu Y Hagerman RJ. Clinical neurological phenotype of FXTAS In: Tassone F Hall DA, editors. FXTAS, FXPOI, and other Premutation disorders. Cham: Springer International Publishing (2016). 1–24.

[10] Hall DA Hermanson M Dunn E Stebbins G Merkitch D Ouyang B. The Corpus callosum splenium sign in fragile X-associated tremor Ataxia syndrome. Mov Disord Clin Pract. (2017) 4:383–8. doi: 10.1002/mdc3.12449, PMID: 30363360 PMC6174407

[11] Loesch DZ Tassone F Atkinson A Stimpson P Trost N Pountney DL. Differential progression of motor dysfunction between male and female fragile X Premutation carriers reveals novel aspects of sex-specific neural involvement. Front Mol Biosci. (2021) 7:577246. doi: 10.3389/fmolb.2020.577246, PMID: 33511153 PMC7835843

[12] Chonchaiya W Utari A Pereira GM Tassone F Hessl D Hagerman RJ. Broad clinical involvement in a family affected by the fragile X premutation. J Dev Behav Pediatr. (2009) 30:544–51. doi: 10.1097/DBP.0b013e3181c35f25, PMID: 19996900 PMC2822648

[13] Coffey SM Cook K Tartaglia N Tassone F Nguyen DV Pan R. Expanded clinical phenotype of women with the FMR1 premutation. Am J Med Genet A. (2008) 146A:1009–16. doi: 10.1002/ajmg.a.32060 18348275 PMC2888464

[14] Fay-Karmon T Hassin-Baer S. The spectrum of tremor among carriers of the FMR1 premutation with or without the fragile X-associated tremor/ataxia syndrome (FXTAS). Parkinsonism Relat Disord. (2019) 65:32–8. doi: 10.1016/j.parkreldis.2019.05.010 31126791

[15] Tassone F Protic D Allen EG Archibald AD Baud A Brown TW. Insight and recommendations for fragile X-Premutation-associated conditions from the fifth international conference on FMR1 Premutation. Cells. (2023) 12:2330. doi: 10.3390/cells12182330, PMID: 37759552 PMC10529056

[16] Tassone F Pan R Amiri K Taylor AK Hagerman PJ. A rapid polymerase chain reaction-based screening method for identification of all expanded alleles of the fragile X (FMR1) gene in newborn and high-risk populations. J Mol Diagn. (2008) 10:43–9. doi: 10.2353/jmoldx.2008.070073, PMID: 18165273 PMC2175542

[17] Filipovic-Sadic S Sah S Chen L Krosting J Sekinger E Zhang W. A novel FMR1 PCR method for the routine detection of low abundance expanded alleles and full mutations in fragile X syndrome. Clin Chem. (2010) 56:399–408. doi: 10.1373/clinchem.2009.136101, PMID: 20056738 PMC4031651

[18] Fahn S Tolosa E Marin C. Clinical rating scale for tremor In: Jankovic J Tolosa E, editors. Parkinson's disease and movement disorders. Baltimore: Williams & Wilkins (1993). 271–80.

[19] Trouillas P Takayanagi T Hallett M Currier RD Subramony SH Wessel K. International cooperative Ataxia rating scale for pharmacological assessment of the cerebellar syndrome. The Ataxia neuropharmacology Committee of the World Federation of neurology. J Neurol Sci. (1997) 145:205–11. doi: 10.1016/S0022-510X(96)00231-6, PMID: 9094050

[20] Smith A. Symbol digit modalities test. Los Angeles, CA: Western Psychological Services (1982).

[21] Tombaugh TN. Trail making test a and B: normative data stratified by age and education. Arch Clin Neuropsychol. (2004) 19:203–14. doi: 10.1016/S0887-6177(03)00039-8, PMID: 15010086

[22] Wechsler D. Wechsler Adult Intelligence Scale. San Antonio: The Psychological Corporation (1997).

[23] Storey E Bui MQ Stimpson P Tassone F Atkinson A Loesch DZ. Relationships between motor scores and cognitive functioning in FMR1 female premutation X carriers indicate early involvement of cerebello-cerebral pathways. Cerebellum Ataxias. (2021) 8:15. doi: 10.1186/s40673-021-00138-0, PMID: 34116720 PMC8196444

[24] Hocking DR Loesch DZ Stimpson P Tassone F Atkinson A Storey E. Delineating the relationships between motor, cognitive-executive and psychiatric symptoms in female FMR1 Premutation carriers. Front Psych. (2021) 12:742929. doi: 10.3389/fpsyt.2021.742929, PMID: 34925088 PMC8678043

[25] Fitzpatrick LE Jackson M Crowe SF. Characterization of cerebellar ataxia in chronic alcoholics using the international cooperative Ataxia rating scale (ICARS). Alcohol Clin Exp Res. (2012) 36:1942–51. doi: 10.1111/j.1530-0277.2012.01821.x, PMID: 22568470

[26] Hall DA Loesch D Svymbersky T Berry-Kravis E Ouyang B. Essential tremor-like Phenotype in Fragile X Carrier Women. Department of Neurological Sciences, Rush University Medical Centre. [Manuscript submitted for publication]., PMID: 34925088

[27] Grigsby J Cornish K Hocking D Kraan C Olichney JM Rivera SM. The cognitive neuropsychological phenotype of carriers of the FMR1 premutation. J Neurodev Disord. (2014) 6:28. doi: 10.1186/1866-1955-6-28, PMID: 25136377 PMC4135346

[28] Famula J Ferrer E Hagerman RJ Tassone F Schneider A Rivera SM. Neuropsychological changes in FMR1 premutation carriers and onset of fragile X-associated tremor/ataxia syndrome. J Neurodev Disord. (2022) 14:23. doi: 10.1186/s11689-022-09436-y, PMID: 35321639 PMC8942145

[29] Hocking DR Loesch DZ Stimpson P Tassone F Atkinson A Storey E. Relationships of motor changes with cognitive and neuropsychiatric features in FMR1 male carriers affected with fragile X-associated tremor/Ataxia syndrome. Brain Sci. (2022) 12:1549. doi: 10.3390/brainsci12111549, PMID: 36421873 PMC9688438

[30] Tassone F Greco CM Hunsaker MR Seritan AL Berman RF Gane LW. Neuropathological, clinical and molecular pathology in female fragile X premutation carriers with and without FXTAS. Genes Brain Behav. (2012) 11:577–85. doi: 10.1111/j.1601-183X.2012.00779.x, PMID: 22463693 PMC3965773

